# Microfluidic-Based Continuous Fabrication of Ultrathin Hydrogel Films with Controllable Thickness

**DOI:** 10.3390/polym15132905

**Published:** 2023-06-30

**Authors:** Xiaozhi Ouyang, Cheng Huang, Sha Cheng, Pengchao Zhang, Wen Chen

**Affiliations:** 1 Key Laboratory of Advanced Technology for Materials Synthesis and Processing, School of Materials Science and Engineering, Wuhan University of Technology, Wuhan 430070, China; 2 Hubei Longzhong Laboratory, Wuhan University of Technology Xiangyang Demonstration Zone, Xiangyang 441000, China; 3 Sanya Science and Education Innovation Park, Wuhan University of Technology, Sanya 572024, China

**Keywords:** microfluidic, laminar flow, ultrathin hydrogel film, continuous fabrication, controlled thickness

## Abstract

Ultrathin hydrogel films composed of cross-linked polymer networks swollen by water, with soft and moisturized features similar to biological tissue, play a vital role in flexible biosensors and wearable electronics. However, achieving efficient and continuous fabrication of such films remains a challenge. Here, we present a microfluidic-based strategy for the continuous fabrication of free-standing ultrathin hydrogel films by using laminar flow, which can be precisely controlled in the micrometer scale. Compared with conventional methods, the microfluidic-based method shows advantages in producing hydrogel films with a high homogeneity as well as maintaining the structural integrity, without the need of supporting substrates and sophisticated equipment. This strategy allows the precise control over the thickness of the hydrogel films ranging from 15 ± 0.2 to 39 ± 0.5 μm, by adjusting the height of the microfluidic channels, with predictable opportunities for scaling up. Therefore, our strategy provides a facile route to produce advanced thin polymer films in a universal, steerable, and scalable manner and will promote the applications of thin polymer films in biosensors and wearable electronics.

## 1. Introduction

The stark difference between soft tissues and rigid electronics makes it difficult for conventional electronic devices to achieve seamless interfaces with biological tissues and thus limits their applications in biosensors and wearable electronic [1,2,3,4]. Ultrathin polymeric films with a thickness down to micrometers, excellent flexibility, and mechanical compliance have arisen as important components to breakthrough the limitations of conventional rigid and bulky devices towards imperceptible and wearable system [5,6,7,8]. Despite the significant potential of ultrathin polymeric films in reducing the device thickness and maintaining high wearable performance, there remains certain limitations. One critical issue is that soft tissue generally has a coarse, irregular surface [9], such as microscale lines and folds on the skin surface, and commonly used, thin polymeric substrates for flexible devices, such as polyethylene terephthalate (PET) [10,11,12], polyvinylidene fluoride (PVDF) [13,14,15], and polyimide (PI) [16,17,18], cannot completely fit the microscopic irregular landscape. This induces the air gap between the devices and skin surface, hampering the formation of ideal skin–device interface and limiting the precise monitoring of bio-electric signals [19,20,21,22].

Hydrogels composed of a three-dimensional cross-linked polymer networks entrapped with water phase, with soft and moisturize features similar to biological tissue, have long been studied for bioprocessing and tissue engineering [23,24,25,26]. Their biological properties offer hydrogels tremendous application in flexible biosensors and wearable electronic [27,28,29,30]. Recently, Kim et al. reported a layer of functionalized hydrogel with a thickness of ≈150 μm, which was used to improve the air gap between wearable devices and human skin into an intimate tissue-like interface, resulting in the excellent performance of wearable bioelectronics [31]. Despite promising potentiality of applying hydrogel film to form an ideal tissue–device interface, the production of ultrathin hydrogel films with controlled thickness and high homogeneity is still a challenging task.

Hydrogel films fabricated by traditional strategies, for instance cast molding [32,33,34] and spin coating [35,36], often had a thickness over hundreds of micrometers and uncontrolled uniformity over a large scale. Spin coating is a widely used and quite efficient method to produce thin films with a controlled thickness down to tens of micrometers. However, the utilization of hydrophilic substrates leads to strong adhesion between the produced hydrogel films and substrates, making it difficult to separate the hydrogel thin films. The freezing microtomy is effective in producing ultrathin hydrogel films, but the cost is substantial [37,38]. Very recently, a novel cold lamination method was demonstrated to fabricate ultrathin hydrogel films, achieving high homogeneity in thickness across large lateral scales, despite the supporting substrates and sophisticated equipment [39]. In a word, there is still a lack of effective approaches to fabricate ultrathin hydrogel films in a universal, steerable, and scalable manner.

Microfluidic technology can be used to precisely manipulate laminar flow in microscale channels, providing an effective method for the continuous and scalable fabrication of ultrathin hydrogel films. Until now, several kinds of hydrogel films have been prepared by using appropriate film forming methods. For example, Callahan’s team prepared heterogeneous hydrogel films with bioactivity gradients in microfluidic channels by controlling the flow rate of hydrogel prepolymers and using UV-induced polymerization [40] Heterogeneous polyethylene glycol dimethacrylate and polyethylene glycol-based hydrogel films were prepared with concentration gradients of multiple peptides, and cells were introduced into heterogeneous hydrogels, which could be used for cell culture and tissue engineering [41,42]. In addition to photopolymerization, polymer films can also be obtained through films formation methods such as ion crosslinking and non-solvent induced phase separation (NIPS) [43]. For example, Gao’s group developed a wet- spinning method to continuously fabricate bioinspired graphene oxide (GO) hydrogel films by ionic crosslinking [44]. These works demonstrated the feasibility of the development of polymer films based on microfluidics laminar flow. However, this area is still in its infant stage, and many issues need to be addressed. For example, how to precisely control the thickness of polymer films, especially free-standing hydrogel films, is still challenging.

In this work, we report a facile scalable fabrication strategy of free-standing ultrathin hydrogel films based on microfluidic-based laminar flow. Compared with conventional methods, the microfluidic-based laminar flow method shows an advantage in continuously producing ultrathin hydrogel films and realizing a high homogeneity across large scales, without the need of supporting substrates and sophisticated equipment. More importantly, the thickness of hydrogel films can be precisely controlled by easy designing the height of microfluidic laminar flow, in this instance down to 15 μm in thickness, with predictable opportunities for scaling up. Besides the classical chemical-cross-linked hydrogels, the conventional polymer films by NIPS are also demonstrated in this work. For instance, the classical thin polymer film including thin PVDF and PI films were fabricated. Thus, our work provides a simple approach for developing advanced ultrathin polymer films and will promote foreseeable application in flexible electronics.

## 2. Materials and Methods

### 2.1. Materials

Sodium alginate (SA), propylene glycol methyl ether acetate (PGMEA, containing 50 ppm BHT stabilizer), isopropyl alcohol, and fluorescein sodium salt were purchased from Aladdin. Polyimide (PI), polydimethylsiloxane (PDMS) elastomer (Sylgard 184, Dow Corning, Midland, MI, USA), and 1-Methyl-2-pyridyl ketone (NMP) were purchased from Macklin Biochemical Co., Ltd. (Shanghai, China). N,N-dimethylformamide (DMF), calcium chloride (CaCl_2_), trimethylchlorosilane (TMCS), and ethanol were purchased from Sinopharm Chemical Reagent Co., Ltd. (Shanghai, China). Photoresist (SU-8 3025, 3050) was purchased from Kayaku Advanced Materials, Inc. (Westborough, MA, USA). An Aquapro ultrapure water system was used for deionized water. All the reagents were used without further purification.

### 2.2. Preparation

Microfluidic devices were fabricated using standard photolithography and molding processes [45,46]. Briefly, a photoresist film was prepared on a silicon wafer using spin coating method. The film was baked at 65 °C (soft bake) and 95 °C (hard bake) for 1 min and 10 min, respectively, and then cooled to 25 °C. The silicon wafer with photoresist film, together with the photomask with predesigned patterns, was irradiated with UV light at 7.3 mJ cm^−2^ for 14 s. The UV irradiation time was determined according to the thickness of the photoresist. In this work, the UV irradiation time was 13, 15, and 19 s for the photoresist films with thickness of 15, 20, and 40 μm, respectively. Then, the silicon wafer was baked at 95 °C for 3 min, respectively. After being cooled to 25 °C and development, the patterned wafer was fabricated and served as the mold. The surface of the template was coated with TMCS for 30 min to make the surface hydrophobic. The PDMS mixture was cast on the template and solidified at 80 °C for 30 min to obtain the PDMS replica. The microfluidic devices were obtained by bonding the PDMS replicas to plasma-treated glass substrates.

Preparation of calcium alginate (CA) hydrogel films: Some 1.3 wt% SA solution was injected into the microfluidic devices at a flow rate of 300 μL/min and exposed to the coagulating bath (1 mol L^−1^ CaCl_2_). CA hydrogel films were obtained via in situ Ca^2+^ cross-linking. The prepared hydrogel films were washed by immersing in the excess distilled water and then dried at room temperature.

Preparation of PVDF films: PVDF powder (*M_w_* = 600,000) was dissolved in DMF by an ultrasonic treatment for 30 min to obtain a 13 wt% PVDF solution, which was injected into the microfluidic device at a flow rate of 300 μL/min and solidified in the coagulating bath composed of distilled water by using non-solvent induced phase separation.

Preparation of PI films: PI solution (6 wt%) was obtained by dissolving PI powders in NMP. Porous PI films were fabricated by using the microfluidic-based continuous printing strategy at a flow rate of 300 μL min^−1^ in the coagulating bath (distilled water) via non-solvent induced phase separation.

Preparation of CA hydrogel films by the blade casting method [18,47]: A 1.3 wt% SA solution was cast onto a glass surface with a scraper (height: 50 μm), which was then soaked in a coagulation bath (1 mol L^−1^ CaCl_2_) for 1 min to obtain the CA hydrogel films. The prepared hydrogel films were washed by immersing in excess distilled water and then dried at 25 °C.

### 2.3. Instruments and Characterization

An ultraviolet lithography machine (7.3 mJ cm^−2^, URE-2000A, Institute of Optics and Electronics, Chinese Academy of Sciences, Chengdu, China) was used to generate UV light for the fabrication of the templates with desired structures. The thickness of the CA hydrogel films was characterized by using the inverted fluorescence microscope (Axiolab 5, ZEISS, Oberkochen, Germany). The surface morphology and thickness of the films were characterized by using field emission scanning electron microscope (FESEM, JEOL-7100F, Tokyo, Japan). The mechanical properties of CA, PI, and PVDF films were characterized by an advanced tensile/compressive testing machine (F105-IM, Mark-10, New York, NY, USA). The transmittance of the ultrathin films with different thicknesses was characterized by UV–visible absorption spectroscope (UV-2550, Shimadzu, Kyoto, Japan). The pore size distribution of the PI and PVDF films were characterized by using the comprehensive membrane pore size analyzer (BSD-PB, Beishide Instrument Technology, Co., Ltd., Beijing, China).

## 3. Results and Discussion

**Figure 1:**
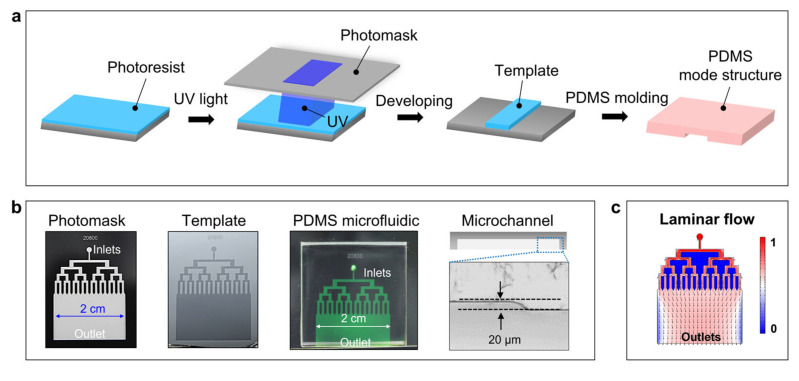
Microfluidic device fabricated by soft lithography and replica techniques. (**a**) Schematic illustration of the lithography and replica processes. (**b**) Optical images that show the photomask, the template prepared by lithography, PDMS microfluidic device, and the microchannel. (**c**) Numerical simulation of the flow direction profiles in the microchannels. A small amount of dye (Sodium fluorescein: 0.02 g/L) was used for clear observation.

Microfluidic technology enables the precise manipulation of laminar flow in the micrometer scale, which is beneficial to the development of thin polymer films [48,49,50]. To generate the laminar flow, microfluidic devices with specific microchannels were designed by using AutoCAD (2020, Autodesk, San Francisco, CA, USA) and were fabricated via soft lithography and replica techniques (Figure 1a). In this work, we designed and fabricated the microfluidic devices with hierarchical tree-like microchannels for the generation of laminar flow and the width of microchannel outlets in the centimeter scale for the preparation of ultrathin polymer films. Figure 1b shows the designed photomask and the relevant template prepared by lithography. The microfluidic chip was formed by PDMS [51] cast molding processes and then bonded onto a plasma-treated glass substrate (Figure 1b). In addition, considering the excessive aspect ratios as well as the soft property of PDMS, a rigid support sheet (in this work the cover glass was used) was introduced to the PDMS matrix to prevent the deformation of the microchannels, which would lead to the inhomogeneous thicknesses of the microfluidic laminar flow and the obtained polymer films.

To verify whether the liquid flow in the fabricated microchannels is laminar flow (i.e., the same velocity and direction of the liquid flow), we first calculated the Reynolds number (Re) according to the following Equation (1) [52,53,54]:

Re = ρvd/μ(1)

where ρ, v, and μ are the density, velocity, and viscosity coefficient of the polymer solutions, respectively, and d is the height of the microchannels. According to the value of Re, the liquid flow can be divided into laminar flow and turbulent flow [55,56,57,58]. At low Re (i.e., Re < 2000), the liquid flow in the microchannels is laminar flow. Otherwise, the liquid flow is turbulent flow. In this work, the Re in the microchannels was 0.00077 ± 0.00005 (Table 1), indicating the liquid flow was laminar flow. In addition, the numerical simulation further demonstrated the successful formation of laminar flow in the microchannels (Figure 1c).

**Figure 2:**
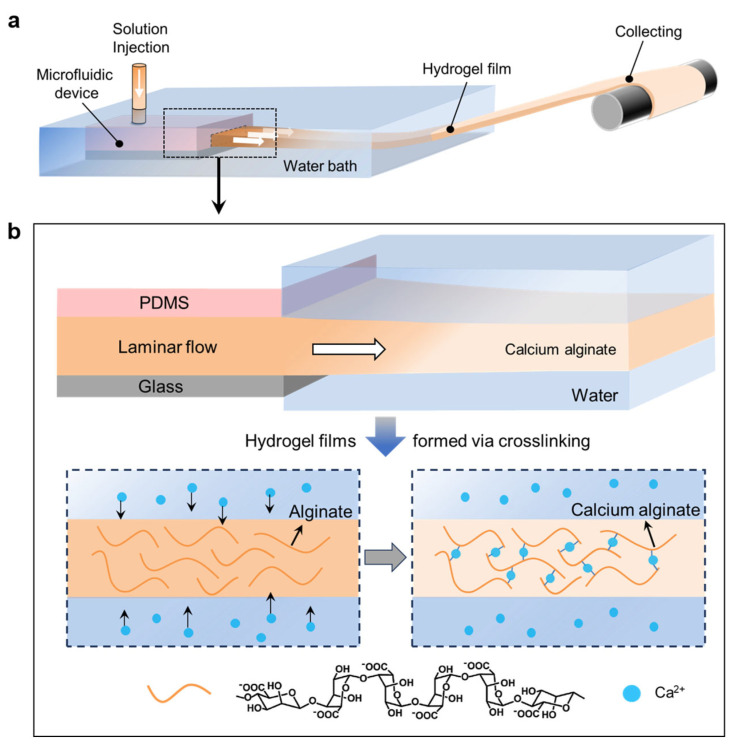
(**a**) Schematic of the continuous fabrication of thin CA hydrogel films with steps involving laminar flow generated in the microchannels, film-formation in the water bath, and collecting. (**b**) The crosslinking of alginate by Ca^2+^ ions to convert the laminar flow into the CA hydrogel film.

**Figure 3:**
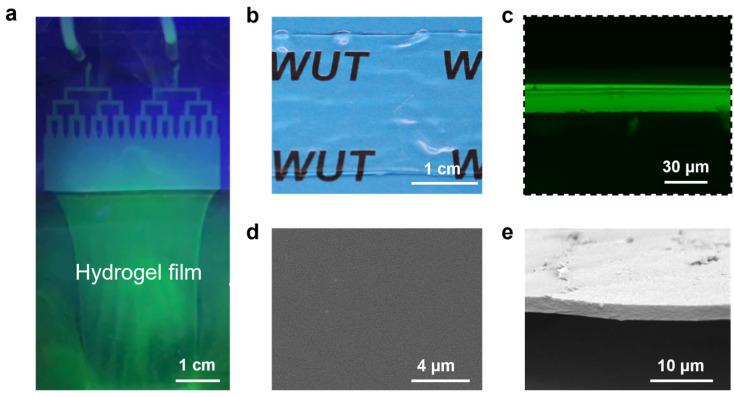
The continuous fabrication and morphological characterization of CA hydrogel film. (**a**) Optical photograph of the microfluidic-based continuous production of CA hydrogel films. (**b**) Optical images, (**c**) cross-sectional fluorescent image, (**d**) surface, and (**e**) cross-sectional SEM images of the CA hydrogel film. A small amount of dye (Fluorescein sodium salt: 0.02 g/L) is used here for clear observation.

In this study, a reaction solution containing SA was used to demonstrate the applicability of our microfluidic-based laminar flow strategy. With appropriate flow rate, the solution from the syringe was injected into the microfluidic device and printed out as a laminar flow of the reaction solution into the coagulating bath (Figure 2a). Meanwhile, Ca^2+^ ions in the coagulating bath, which was previously dissolved with CaCl_2_, diffused into the laminar flow of the reaction solution (Figure 2b). Through electrostatic interaction, Ca^2+^ ions can replace the binding sites of Na^+^ ions in the chain segment; that is, Ca^2+^ ions and carboxyl groups in alginate form “egg-box” structures, leading to the sol-gel transformation of the reaction solution [59,60]. Thus, the laminar flow was immediately converted into the CA hydrogel film. This CA hydrogel film could be continuously printed by solution injecting through microfluidic devices and then be collected without the need of support substrates (Figure 3a and Video S1). This facilitates the formation of hydrogel film into a free-standing state, which is especially critical for maintaining the structural integrity of ultrathin samples (Figure 3b). The cross-sectional fluorescent image of the hydrogel (Figure 3c) indicated the film thickness of 19 μm. After the gel was dried, the clean cross-sectional and smooth surface morphology as revealed by the scanning electron microscope (SEM) image of a free-standing hydrogel film suggest a high homogeneity of the produced samples (Figure 3d,e).

**Figure 4:**
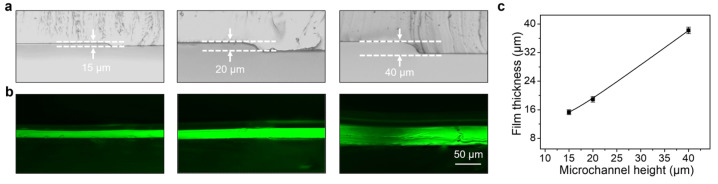
Control over the thickness of the CA hydrogel films. (**a**) Optical images of microfluidic devices with different microchannel heights. (**b**) Cross-sectional fluorescent image of the CA hydrogel prepared by microfluidic devices with different microchannel heights. (**c**) The correlation between the thickness of the produced CA hydrogel films and the microchannel heights.

The thickness of the microfluidic laminar flow is adjustable (through controlling the height of microchannels, which was determined by the photoresist; see details in Section 2.2), thus allowing the controlled thickness of the fabricated hydrogel films. To demonstrate this, we prepared various microfluidic devices in thickness range from 15 to 40 μm (Figure 4a). Experiment results indicated the consequent thickness of the produced CA hydrogel films increased from 15 ± 0.2 to 39 ± 0.5 μm along with the increase of the microchannel height (Figure 4b,c). The thickness of the prepared hydrogel films was almost same as the thickness of the microfluidic channels, which can be attributed to rapid sol-gel transformation of the laminar flow by Ca^2+^ ions induced crosslinking. Therefore, by adjusting the thickness of photoresist, the height of the microchannels was easily and accurately regulated in the micrometer scale, showing advantage in realizing the precisely control in thickness of the liquid flow as well as the formed hydrogel films.

**Figure 5:**
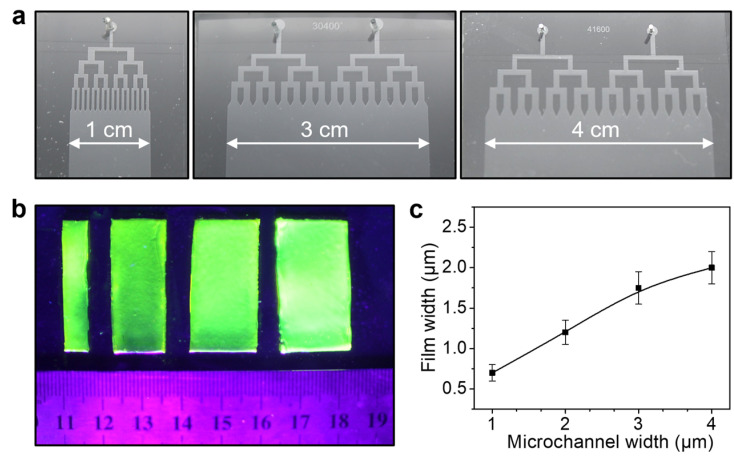
Control over the width of the ultrathin CA hydrogel films. (**a**) Optical images of microfluidic devices with different microchannel width. (**b**) Fluorescent image of the CA hydrogel films prepared by microfluidic devices with different microchannel width. (**c**) The correlation between the width of the produced CA hydrogel films and the microchannel width.

Moreover, the width of the microfluidic laminar flow can also be regulated by extending the width of microchannels, which was determined by initial designed microchannel patterns on photomask. For demonstration, three kinds of microfluidic devices with different widths were prepared, as shown in Figure 5a. Accordingly, the width of hydrogel films increased from 0.7 ± 0.1 to 2.2 ± 0.1 cm along with the increasing of the microchannel width (Figure 5b). It should be noted that the width of the prepared hydrogel films was slightly smaller than that of the microchannels (Figure 5c). This can be attributed to the fact that the velocity of the laminar flow near the edge of the microchannels was close to zero, thus narrowing the width of the laminar flow. In spite of this, the large-area thin hydrogel films were readily producible by further increasing the width of microchannels, with greater opportunity for practical applications.

**Figure 6:**
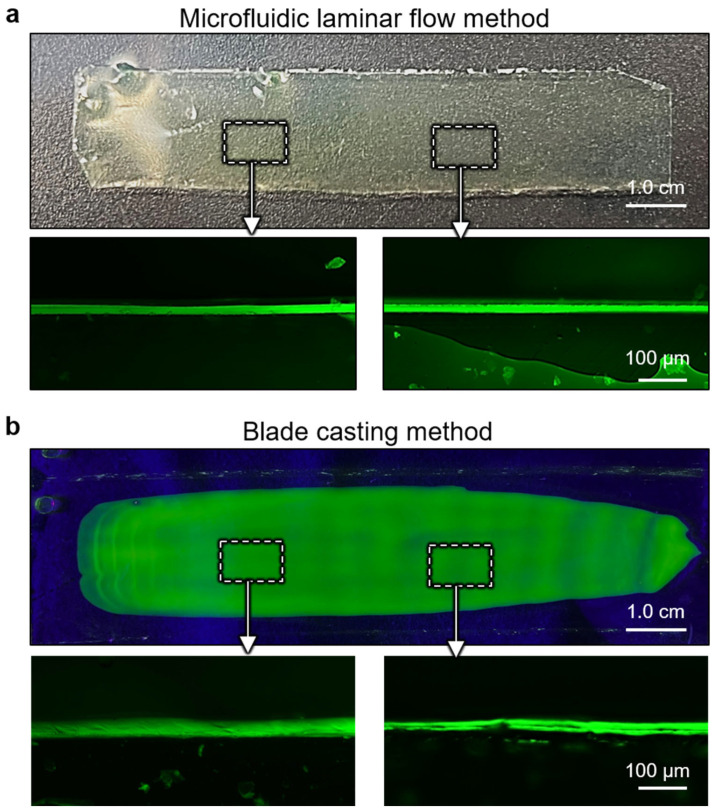
Comparison of uniformity of the ultrathin CA hydrogel films prepared by microfluidic laminar flow method and blade casting method. Optical images of surface and cross-section for hydrogel film prepared by (**a**) microfluidic laminar flow method and (**b**) blade casting method.

Figure 6a shows the optical image of a 10 cm-long ultrathin CA hydrogel film with a width of 2 cm, prepared by the microfluidic laminar flow method. As comparison, CA hydrogel films were also prepared by a traditional blade casting method (Figure 6b). The cross-sectional fluorescent images of the CA hydrogel film at different positions identify the uneven surface of the as-prepared hydrogel films by blade casting method. In contrast, the hydrogel films produced by the microfluidic laminar flow method exhibit a remarkable advantage in precise control of the film thickness with high homogeneity. Moreover, benefitting from the in situ cross-linking of microfluidic laminar flow to form free-standing films, the structural integrity of hydrogel films can be maintained to the maximum extent. The process was quite difficult for conventional approach, as the produced hydrogel films need to be delaminated from support substrate into a free-standing state, which generally leads to the destruction of the films, especially for ultrathin samples. Therefore, the microfluidic laminar flow method not only shows precisely controlling in film thickness with a high homogeneity but also exhibits advantage in maintaining structural integrity, which is crucial for ultrathin hydrogel film, thus superior to conventional approaches.

**Figure 7:**
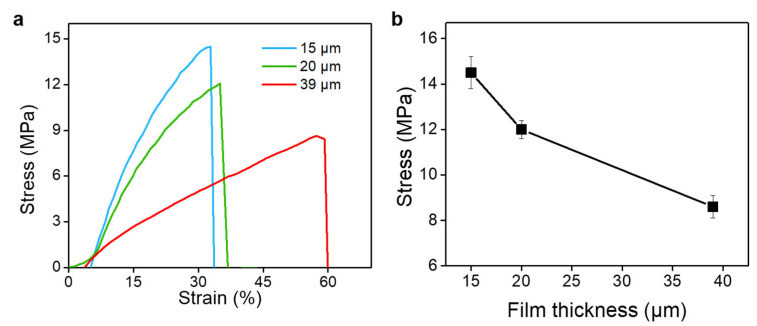
Mechanical properties of the ultrathin CA hydrogel films. (**a**) Typical stress–strain curves of the ultrathin CA hydrogel films prepared by microfluidic laminar flow method. (**b**) The relationship between the strength and thickness of the CA hydrogel films.

The mechanical properties of the ultrathin CA hydrogel films prepared by the microfluidic laminar flow method were evaluated. Figure 7a shows the typical stress–strain curves of the ultrathin CA hydrogel films with a series of thickness. The tensile strength of the CA hydrogel films increases when their thickness decreases from 39 μm (8.6 ± 0.5 MPa) to 15 μm (14.5 ± 0.7 MPa) (Figure 7b). We speculated that this result might be attributed to the insufficient crosslinking for the thicker hydrogel films. In this work, the ionic crosslinking time for preparing CA hydrogel is about 20 s, which is much lower than the reported crosslinking time (several minutes) in the literature [61,62]. By increasing the crosslinking time from 20 s to 20 min, the tensile strength of the CA hydrogel film with thickness of 39 μm increased 8.6 ± 0.5 MPa to 14.7 ± 0.7 MPa, which was similar with the one with thickness of 15 μm (Figure S1). Due to the prolonged immersion time, Ca^2+^ ions can effectively diffuse into the thick hydrogel films, leading to enough crosslinking and increased mechanical properties. These results demonstrate that the mechanical properties can be regulated by controlling the crosslinking of the hydrogel films.

**Figure 8:**
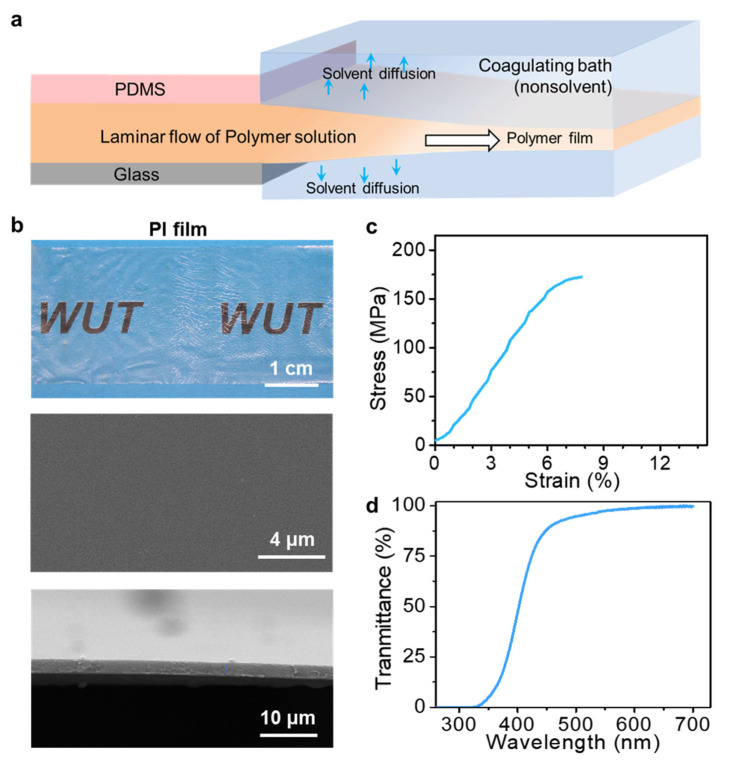
Fabrication of thin polymer films by microfluidic laminar flow method via the NIPS process. (**a**) Schematic of the fabrication of ultrathin polymer films by NIPS with steps involving extrusion of laminar solution flow through the microchannels of microfluidic device and the subsequent phase separation induced by solvent diffusing into the water bath. (**b**) Optical and SEM images of PI films prepared by microfluidic laminar flow method via the NIPS process. (**c**) Typical stress–strain curve and (**d**) transmittance of the PI film.

In addition to the classical chemical-cross-linked hydrogels, the polymer films formed by non-solvent induced phase separation (NIPS) were also investigated by the microfluidic laminar flow method. Figure 8a presents the schematic illustration of the fabrication of thin polymer films through the NIPS process. It included the extrusion of the microfluidic laminar flow of polymer solutions and the solidification of microfluidic laminar flow in the coagulating bath through NIPS. The extrusion solutions were composed of polymer dissolved in the water-miscible solvent. The coagulating bath contained non-solvent water. The fabrication process began with injection of polymer solution into water. When a solution laminar flow was extruded from microfluidic channels to touch the water bath, phase separation of the solution layer occurs. Water-soluble solvent was diffused into the water bath, whereas the water-insoluble polymer stayed in laminar flow with increased phase density and ultimately solidified into thin polymer films.

**Figure 9:**
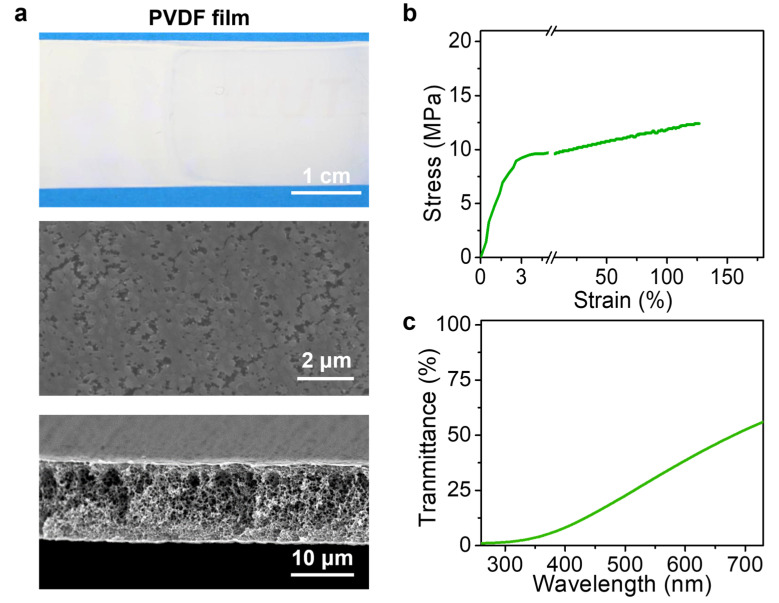
(**a**) Optical and SEM images of PVDF films prepared by microfluidic laminar flow method via NIPS process. (**b**) Typical stress–strain curve and (**c**) transmittance of the PVDF film.

This process can be applied to various polymers. The representative cases using PVDF in DMF and PI in NMP are demonstrated in this work. Compared with the hydrogel films prepared by Ca^2+^ ions induced crosslinking, the polymer films prepared by NIPS were observed to have thinner thickness than that of the microfluidic channels, as shown in Figure 8b and Figure 9a. This observation can be attributed to the diffusion of the solvent in the laminar flow into water bath during the NIPS process, which induced the phase separation of polymer and thus formed polymer films with thinner thickness than that of microfluidic channels. Figure 8b shows the optical image of PI film with smooth surfaces prepared by the microfluidic laminar flow method through the NIPS process (Video S2). The cross-sectional and surface morphology were revealed by SEM images, further indicating the smooth surface with high homogeneity and its ultrathin feature in thickness. In addition, the mechanical properties of the as-obtained polymer films were also evaluated. Figure 8c shows the typical stress–strain curve of the PI films prepared by microfluidic laminar flow method. The tensile strength of the PI films with thickness of 2 ± 0.2 μm was 170 ± 20 MPa. The relatively high tensile strength can be attributed to its dense structure, which was demonstrated by the SEM images shown in Figure 8b and the characterization of the pore size distribution of the PI film (Figure S2). Moreover, due to the dense structures, the as-prepared PI films show a transparent property. This feature was demonstrated by optical image in Figure 8b as the PI film is transparent to the covered pattern. The relevant light transmission property of the PI films was further confirmed by ultraviolet–visible (UV–vis) spectra, which indicated that the as-prepared PI films had a good transparency in the visible light range, as shown in Figure 8d.

PVDF films with smooth surfaces in high homogeneity were also achieved by the microfluidic laminar flow method via NIPS (Video S3). In contrast to the PI films with dense structures, the as-obtained PVDF films possessed an obvious loose morphology with porous structures (Figure 9a). The pore size distribution testing result indicated that the pore size of the PVDF films was 150 ± 11 nm (Figure S3). This structure feature resulted in a relatively low tensile strength (Figure 9b). The PVDF films exhibited white opacity, which completely blocked the covered pattern view and showed a low light transmittance, as demonstrated by the UV–vis spectra (Figure 9c). In conclusion, the microfluidic laminar flow method was further demonstrated to be universal to conventional polymer films formed by NIPS, whose structure morphology with high uniformity can be achieved as well. The results further indicated the universality of the microfluidic laminar flow method for fabricating advanced polymer films.

## 4. Conclusions

In this work, free-standing ultrathin hydrogel films were facilely produced by developing a microfluidic laminar flow method. Compared with conventional methods, the microfluidic laminar flow method shows advantages in producing hydrogel films with high homogeneity as well as maintaining the structural integrity, without the need of supporting substrates and sophisticated equipment. More importantly, the width and thickness of the hydrogel films can be precisely controlled by designing the width and height of microfluidic channels, with predictable opportunities for scaling up. Beside the classical chemical-cross-linked hydrogels, polymer films formed by NIPS were also feasible to the microfluidic laminar flow method. Therefore, our strategy provides a facile route to produce advanced thin polymer film in a universal, steerable, and scalable manner and will promote the applications of thin polymer films in biosensors, wearable electronics, and many other applications.

## Figures and Tables

**Table 1:** Calculated results of reaction solution Re in microchannel.

Re	μ (mPa.s)	ρ (kg/m^3^)	v (m/s)	d (μm)
0.00082	600	1.2	0.0046	90
0.00076	597	1.1	0.0046	90
0.00075	601	1.1	0.0046	90

## Data Availability

The data presented in this study are available from the corresponding author upon reasonable request.

## Supplementary Materials

The following supporting information can be downloaded at: https://www.mdpi.com/article/10.3390/polym15132905/s1, Figure S1: The stress–strain curves of CA hydrogel films with a thickness of 40 μm prepared by microfluidic laminar flow method at different crosslinking times; Figure S2: The pore size distribution of the fabricated PI films; Figure S3: The pore size distribution of the fabricated PVDF films; Videos S1–S3: The continuous fabrication of CA, PI, and PVDF films, respectively.
