# Supplementary material for: Microfluidic-Based Continuous Fabrication of Ultrathin Hydrogel Films with Controllable Thickness
Source: Polymers (Basel). 2023 Jun 30;15(13):2905. doi: 10.3390/polym15132905 (PMC10347089; doi:10.3390/polym15132905)
Supplement: Supplementary file 1 [file polymers-15-02905-s001.zip › polymers-2464958-supplementary-Figures S1-S3.pdf]

Supplementary Materials

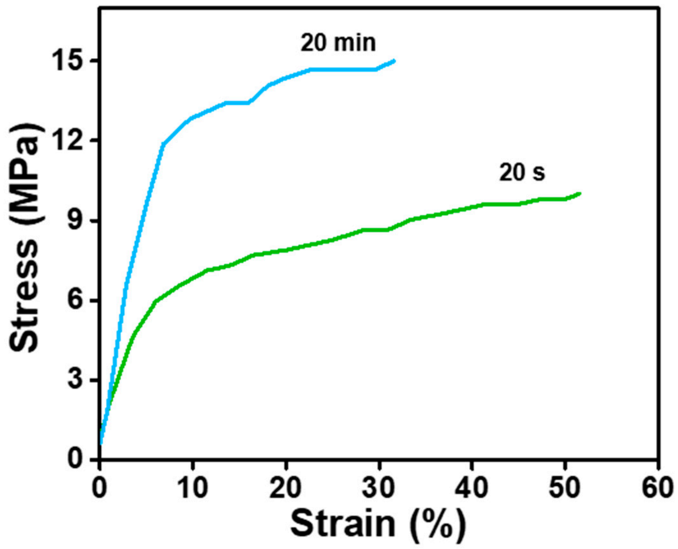

Figure S1. The stress–strain curves of CA hydrogel films with a thickness of 40 μm prepared by microfluidic laminar flow method at different crosslinking times.

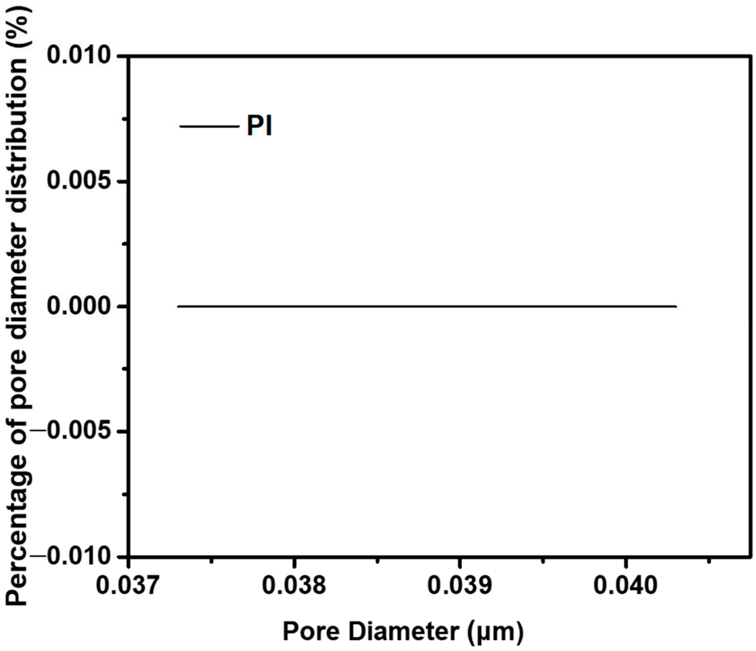

Figure S2. The pore size distribution of the fabricated PI films.

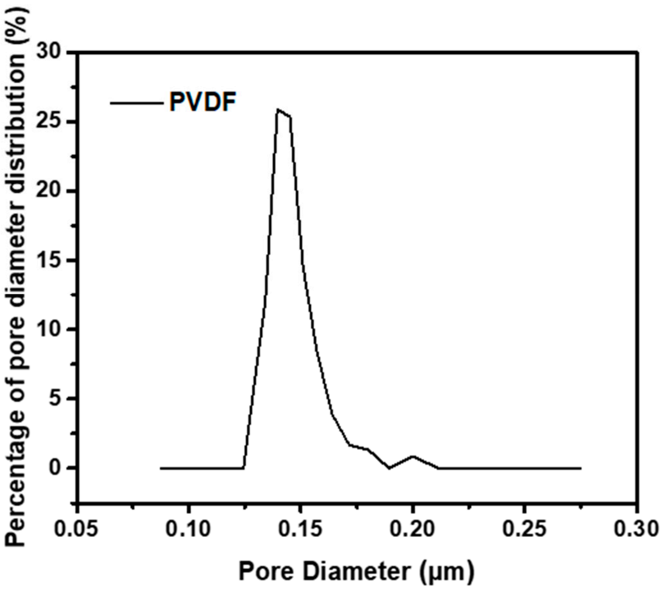

Figure S3. The pore size distribution of the fabricated PVDF films.
